# Dimethylsulfoniopropionate Biosynthetic Bacteria in the Subseafloor Sediments of the South China Sea

**DOI:** 10.3389/fmicb.2021.731524

**Published:** 2021-10-11

**Authors:** Yunhui Zhang, Kai Sun, Chuang Sun, Xiaochong Shi, Jonathan D. Todd, Xiao-Hua Zhang

**Affiliations:** ^1^College of Marine Life Sciences, Frontiers Science Center for Deep Ocean Multispheres and Earth System, Ocean University of China, Qingdao, China; ^2^Laboratory for Marine Ecology and Environmental Science, Qingdao National Laboratory for Marine Science and Technology, Qingdao, China; ^3^Institute of Evolution and Marine Biodiversity, Ocean University of China, Qingdao, China; ^4^School of Biological Sciences, University of East Anglia, Norwich, United Kingdom

**Keywords:** DMSP biosynthesis, subseafloor, South China Sea, bacteria, incubation experiment

## Abstract

Dimethylsulfoniopropionate (DMSP) is one of Earth’s most abundant organosulfur molecules, and bacteria in marine sediments have been considered significant producers. However, the vertical profiles of DMSP content and DMSP-producing bacteria in subseafloor sediment have not been described. Here, we used culture-dependent and -independent methods to investigate microbial DMSP production and cycling potential in South China Sea (SCS) sediment. The DMSP content of SCS sediment decreased from 11.25 to 20.90 nmol g^–1^ in the surface to 0.56–2.08 nmol g^–1^ in the bottom layers of 8-m-deep subseafloor sediment cores (*n* = 10). Very few eukaryotic plastid sequences were detected in the sediment, supporting bacteria and not algae as important sediment DMSP producers. Known bacterial DMSP biosynthesis genes (*dsyB* and *mmtN*) were only predicted to be in 0.0007–0.0195% of sediment bacteria, but novel DMSP-producing isolates with potentially unknown DMSP synthesis genes and/or pathways were identified in these sediments, including *Marinobacter* (*Gammaproteobacteria*) and *Erythrobacter* (*Alphaproteobacteria*) sp. The abundance of bacteria with the potential to produce DMSP decreased with sediment depth and was extremely low at 690 cm. Furthermore, distinct DMSP-producing bacterial groups existed in surface and subseafloor sediment samples, and their abundance increased when samples were incubated under conditions known to enrich for DMSP-producing bacteria. Bacterial DMSP catabolic genes were also most abundant in the surface oxic sediments with high DMSP concentrations. This study extends the current knowledge of bacterial DMSP biosynthesis in marine sediments and implies that DMSP biosynthesis is not only confined to the surface oxic sediment zones. It highlights the importance of future work to uncover the DMSP biosynthesis genes/pathways in novel DMSP-producing bacteria.

## Introduction

Dimethylsulfoniopropionate (DMSP) is an organic sulfur compound produced by many marine algae, bacteria, corals, and some plants (Moran and Durham, 2019; Zhang et al., 2019). Organisms produce and accumulate DMSP to mM concentration for its anti-stress properties as osmoprotectant, thermoprotectant, antioxidant, and protectant against hydrostatic pressure, or for its role as a signaling molecule (Kiene et al., 2000; Sunda et al., 2002; Seymour et al., 2010; Gregory et al., 2020; Zheng et al., 2020). Earth’s oceans alone produce petagrams of DMSP annually (Ksionzek et al., 2016), which is thought to support up to 95 and 15% of microbial carbon and sulfur demands, respectively, through DMSP catabolic pathways (Zubkov et al., 2001). The majority of DMSP (∼75% dissolved DMSP) is degraded by bacteria through the demethylation pathway (Kiene and Linn, 2000), which can liberate the volatile organic sulfur compound methanethiol (MeSH) (Moran and Durham, 2019) by the *dmd* gene products. A DMSP cleavage pathway is far less prominent in marine environments, accounting for 10% of microbial catabolism of dissolved DMSP (Reisch et al., 2011). Microbial DMSP lyase enzymes cleave DMSP to liberate the climate active gas dimethylsulfide (DMS) as a co-product with acrylate, hydroxypropionate, or acryloyl-CoA *via* eight diverse *ddd* gene products (DddD, DddL, DddP, DddQ, DddY, DddW, DddK, and DddX) in bacteria and Alma1 in algae (Curson et al., 2011; Alcolombri et al., 2015; Sun et al., 2016; Zhang et al., 2019; Li et al., 2021). Although most DMS is further used by marine microbial communities (Kappler and Schäfer, 2014), 28.1 (17.6–34.4) Tg S per year is emitted into atmosphere annually (Lana et al., 2011), representing the major biogenic flux of sulfur from oceans to the atmosphere, where its oxidation products promote cloud formation and the backscatter of solar radiation (Stefels et al., 2007; Vallina and Simó, 2007; Zhang et al., 2019).

There are three known pathways for DMSP synthesis named after the primary modification of the amino acid methionine (Met) substrate, a Met methylation pathway in plants and bacteria (Kocsis et al., 1998; Liao and Seebeck, 2019; Williams et al., 2019), a Met transamination pathway in algae, corals, and bacteria (Raina et al., 2009; Curson et al., 2017, 2018), and a Met decarboxylation pathway in the dinoflagellate *Crypthecodinium cohnii* (Uchida et al., 1996). The first DMSP synthesis gene identified was *dsyB* encoding the key SAM-dependent methylthiohydroxybutyrate (MTHB) *S*-methyltransferase of the Met transamination pathway in diverse marine *Alpha-* and *Gammaproteobacteria* (Curson et al., 2017; Williams et al., 2019). Most of bacteria containing *dsyB* are in the *Rhodobacterales* order that is abundant in marine environments, but a few homologs are also in the *Rhizobiales*, *Rhodospirillales*, *Bacteroidetes*, and *Actinobacteria* (Curson et al., 2017; Williams et al., 2019). Functional DsyB-like enzymes termed DSYB (with ∼30% amino acid identity) exist in most eukaryotic corals, dinoflagellates, and haptophytes and ∼20% of diatoms with available transcriptome data (Curson et al., 2018). An isoform MTHB *S*-methyltransferase enzyme, termed TpMMT, was identified in the diatom *Thalassiosira pseudonana* (Kageyama et al., 2018). The Met *S*-methyltransferase of a bacteria Met methylation pathway was identified in diverse bacteria spanning diverse *Alphaproteobacteria* and some *Actinobacteria* (Liao and Seebeck, 2019; Williams et al., 2019). *Thalassospira* and *Novosphingobium* are typical DMSP-producing genera with *mmtN* (Williams et al., 2019). With the exception of TpMMT, which has only been studied in *T. pseudonana*, the other *S*-methyltransferase genes in algae and bacteria serve as reliable reporters for DMSP synthesis in diverse marine environments (Curson et al., 2017; Kageyama et al., 2018; Williams et al., 2019; Sun et al., 2020; Zheng et al., 2020). Approximately 0.35% of marine bacteria contain *dsyB* and are predicted to make DMSP, whereas *mmtN* is far less abundant (Williams et al., 2019). This is far less than those bacteria that are predicted to catabolize DMSP *via* demethylation (∼20% of marine bacteria with *dmdA*) or cleavage pathways (∼20% of marine bacteria with a *ddd* gene) (Curson et al., 2018; Williams et al., 2019). However, there are also many bacterial species that produce DMSP that lack *dsyB* or *mmtN* homologs in their genomes, such as *Celeribacter*, *Marinobacter*, and *Erythrobacter* (Williams et al., 2019; Zheng et al., 2020), indicating the existence of novel DMSP biosynthetic or pathways (Curson et al., 2017; Williams et al., 2019; Zheng et al., 2020; Liu et al., 2021).

Dimethylsulfoniopropionate is ubiquitous in the marine euphotic zone at concentrations ranging from 1 to 100 nM in the open oceans (Dacey et al., 1998; Cui et al., 2015; Galí et al., 2015) to several micromolar in phytoplankton blooms (Van Duyl et al., 1998; Speeckaert et al., 2018). Phytoplankton, such as dinoflagellates, haptophytes, and green algae that contain the highest intracellular DMSP levels (Keller et al., 1989; Curson et al., 2018), are believed to be the main oceanic DMSP producers (Zhang et al., 2019). However, recent studies analyzing the prevalence key algal and bacterial DMSP synthesis genes in environmental samples suggest that bacteria also significantly contribute to marine DMSP production (Williams et al., 2019; Song et al., 2020; Sun et al., 2020; Zheng et al., 2020; Liu et al., 2021), especially in aphotic and deep seawater and surface marine sediments where phytoplankton are scarce.

Dimethylsulfoniopropionate content in surface marine sediments can be up to three orders of magnitude higher than most seawater samples, but these levels are reported to decrease with sediment depth and oxygen availability (Zhuang et al., 2017; Wilkening et al., 2019; Williams et al., 2019). For instance, DMSP concentration in surface sediment from saltmarsh ponds reached 128 nmol g^–1^ and reduced to 3.9–9.8 nmol g^–1^ in anoxic sediments, which was much higher than that in the overlying seawater (0.01–0.07 nmol ml^–1^) (Williams et al., 2019). A sizeable amount of the DMSP in such marine sediment has been proposed to result from the microbial biosynthesis in sediments (Williams et al., 2019; Song et al., 2020; Zheng et al., 2020) along with phytodetritus, depending on the water depth (Zhuang et al., 2017). Microbial DMSP catabolic potential seems to be varied between different sediments. In surface saltmarsh sediments, DMSP lyase genes (*dddD*, *dddL*, and *dddP*) were far more abundant than *dsyB* (Williams et al., 2019), but this was reversed in Mariana Trench deep-sea sediment where *dsyB* far outnumbered both *dmdA* and *dddP* (Zheng et al., 2020). So far, studies on microbial DMSP biosynthesis and cycling have largely focused on surface sediment in the comparison to overlying seawater samples (Williams et al., 2019; Zheng et al., 2020). There are no detailed molecular studies investigating the microbes producing and cycling DMSP through the vertical profile of marine subseafloor sediment. Thus, there is limited understanding of DMSP cycling in these important marine sediment environments.

The South China Sea (SCS) is one of the largest marginal seas in the world. The transport processes of river-borne terrigenous inputs form thick organic-rich sediments in the northern slope of the SCS (Liu et al., 2010, 2016). These SCS sediments constitute ideal material for studying microbial community, metabolism, and pivotal biogeochemical processes (Zhao et al., 2017; Graw et al., 2018). However, the DMSP concentration, microbes, and their importance in producing and cycling DMSP in these sediments is unexplored. In a previous study, we obtained 10 sediment cores (6–8 m deep) from the northern slope of the SCS (Zhang et al., 2021), and investigated the vertical profile of microbial abundance and distribution in this region. Here, we quantified the DMSP concentration in the SCS surface and subseafloor sediments, and combined culture-dependent and -independent methods to explore the spatial distribution of DMSP-producing and catabolic bacteria, aiming to reveal (1) the vertical concentration profiles of DMSP in deep-sea sediments, (2) vertical changes of DMSP-producing bacteria groups, and (3) the potential novel DMSP biosynthetic bacteria in the SCS sediments.

## Materials and Methods

### Sampling and Quantification of Dimethylsulfoniopropionate

**FIGURE 1:**
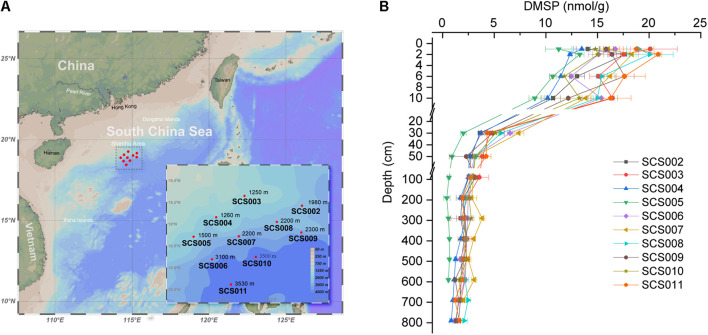
Sitemap **(A)** and DMSP concentrations in the SCS sediments **(B)**. Seafloor depth are indicated in each site. Data are presented as means ± SD.

As described in our previous study (Zhang et al., 2021), 10 sediment gravity cores were collected on the northern slope of the SCS (18.44–19.26°N, 114.25–115.30°E) (Figure 1A), ranging from 6.64 to 8.36 m. The detailed sampling methods and environmental parameters have been reported by Zhang et al. (2021). All 134 sediment samples from 1, 2, 6, 10, 30, 50, 90, 190, 290, 390, 490, 590, 690, and 790 cm depths of these cores have been used to quantify their DMSP content. DMSP was measured as described by Williams et al. (2019). Briefly, 0.1 g of sample was added to a 2-ml glass vial followed by 0.2 ml of 10 M NaOH. The vials were crimped immediately and incubated overnight in the dark at room temperature. The headspace DMS resulting from the alkaline lysis of DMSP was monitored by gas chromatography (GC) with a flame photometric detector (Agilent 7890B GC fitted with a 7693A autosampler) and an HP-INNOWax 30 m × 0.320 mm capillary column (Agilent Technologies J&W Scientific). A calibration curve was produced by alkaline lysis of DMSP standards under the same conditions, and the detection limit for headspace DMS was 0.015 nmol as described by Curson et al. (2017).

### Enrichments for Microbial Dimethylsulfoniopropionate Production

Experiments designed to enrich for DMSP-producing bacteria were carried out according to Liu et al. (2021) with minor modifications. Briefly, to enrich for DMSP-producing bacteria, 0.2 g of sediment samples of SCS008 (1, 30, 50, 90, 390, and 690 cm) was inoculated into 30 ml of modified marine basal medium (MBM) (Zheng et al., 2020) with increased salinity (PSU 50), decreased nitrogen levels (1 mM NH_4_^+^), and 0.5 mM L-Met addition. For the control group, the sediment samples were added in normal MBM (PSU 35, 10 mM NH_4_^+^). The same amount of mixed carbon source (10 mM at a final concentration) was supplemented in normal and modified MBM. Both the control and enrichment assays were carried out in triplicate and incubated at 90 rpm, 16°C. DMSP concentration in incubations was monitored by GC as described above every 7 days. Sample names were assigned with the suffix T0, ENR, and CON for natural (non-incubated) sediments, DMSP-enriched samples, and control samples, respectively.

### Quantification of Dimethylsulfoniopropionate Biosynthetic and Catabolism Genes

Total DNA for qPCR and 16S rRNA gene amplicon sequencing was isolated from 0.25 g of sediment sample as described by Carrión et al. (2017). All qPCR was performed on a StepOnePlusTM Real-Time PCR System (Applied Biosystems, United States) in triplicate. For T0 samples of SCS008 (at 14 depth layers), as well as the ENR and CON samples in the enrichment experiment, the abundance of DMSP biosynthetic gene *dsyB* and *mmtN*, as well as DMSP catabolic gene *dddP*, *dmdA* (C/2), and *dmdA* (D/1) was determined. The primers (5′–3′) used and the annealing temperature were listed in Supplementary Table 1. The PCR reactions were conducted as follows: an initial denaturation at 95°C for 3 min, then 35 cycles of 95°C for 20 s, the corresponding annealing temperature for 30 s, 72°C for 30 s. A melt curve was run after PCR as follows: 95°C denaturation for 1 min, 0.5°C increment from annealing temperature with signal collection. The standard curve making for qPCR was carried out according to the method previously described by Williams et al. (2019).

### Bacterial Isolation and Screening of Dimethylsulfoniopropionate -Producing Isolates

The sediment samples and ENR cultures were serially diluted and spread on Marine Agar (MA) plates and incubated at 28°C for 5–7 days. Single colonies were picked and subsequently purified three times by streaking. The 16S rRNA genes of DMSP-producing isolates were amplified using the primer set 27F/1492R (Langmead and Salzberg, 2012) and sequenced. Taxonomy information identifying these cultivated strains was elucidated from the Ezbiocloud server^1^. Representative strains of each species identified were screened for DMSP synthesis capability by growing them in normal MBM with 0.5 mM Met. Briefly, the isolates were inoculated into 600 μl of MBM and incubated at 28°C, 170 rpm for 24 h, and then 200 μl of the culture was added into the glass vials for DMSP quantification as described above. The presence of known DMSP biosynthetic genes in DMSP-producing isolates was tested by PCR using degenerate primers to *mmtN* and *dsyB* as described by Liu et al. (2021). The neighbor-joining tree based on partial 16S rRNA gene sequences of representative isolates was performed by using the software package MEGA X (Kumar et al., 2018). Genetic distances were calculated by using Kimura’s two-parameter model (Kimura, 1980). The topology of the phylogenetic trees was evaluated by the bootstrap resampling method with 1,000 replicates. The resulting tree was visualized using EvolView (Subramanian et al., 2019).

### 16S rRNA Gene Amplicon Sequencing and Statistical Analysis

The 16S rRNA gene amplicon sequencing of SCS008 (1, 50, 90, 390, and 690 cm) was performed in our previous study (Zhang et al., 2021) and re-analyzed in this study with the enriched samples. The enriched cultures (1 ml) were centrifuged and genomic DNA was extracted from the resulting pellet using the Power Soil DNA Isolation Kit (MoBio Laboratories) and a FastPrep-24 cell disrupter (MP Biomedicals) according to the manufacturer’s instructions. The primer set of 515FmodF (5′-GTGYCAGCMGCCGCGGTAA-3′) and 806RmodR (5′-GGACTACNVGGGTWTCTAAT-3′) was used to amplify the V4 hypervariable region of the 16S rRNA gene from bacteria and archaea (Walters et al., 2016). Polymerase chain reactions (PCR), sequencing on an Illumina Miseq PE300 platform (Illumina, San Diego, CA, United States), and quality control of raw reads were performed by the Majorbio Bio-Pharm Technology Co. Ltd. (Majorbio, Shanghai, China) as described by Zhang et al. (2021). Operational taxonomic units (OTUs) were clustered with a 97% similarity cutoff using uparse (Edgar, 2013) (version 7.0.1090^2^). Singleton OTUs that may represent sequencing errors were removed before downstream analyses. The taxonomy of each 16S rRNA gene sequence was analyzed by Ribosomal Database Project (RDP) Classifier algorithm (version 2.11^3^) (Wang et al., 2007) against the Silva database (Release132^4^). To equalize sequencing depth, each sample was rarefied to 34,350 reads (the lowest sequence number across all samples) for further analysis. The raw data were submitted into the National Center for Biotechnology Information (NCBI) Sequence Read Archive (SRA) database. For beta diversity, principal coordinate analysis (PCoA) plot was performed with the “vegan” package in R (version 3.6.1) using the Bray–Curtis distance matrix. The significant differences between genera in CON and ENR samples were tested by Student’s *t*-test with the function t.test in R. The known DMSP-producing genera were identified according to Williams et al. (2019), Zheng et al. (2020), and the DMSP-producing isolates in the current study. The plastid sequences were analyzed against RDP 16S rRNA database (see text footnote 3).

### Metagenomic Sequencing for Enriched Samples and Analysis

Metagenomic sequencing was performed on samples from the enrichment experiments. Three replicates from the CON or ENR group at 1, 50, 90, and 390 cm were combined, generating eight samples in total. Quality of the extracted DNA was measured by agarose gel electrophoresis and Qubit 3.0 Fluorometer (ThermoFisher). The quality of the library was examined by Agilent 2100 bioanalyzer. Paired-end metagenomic shotgun sequencing was performed on the BGI MGISEQ-2000 platform (insert size, 300–400 bp; read length, 150 bp). All the raw reads containing >10% of undefined bases, >20% of low-quality bases, and that had >15 bases matching the adapters were removed. High-quality short reads of each DNA sample were assembled by the MEGAHIT (Li et al., 2015). Gene prediction was performed using MetaGeneMark (Zhu et al., 2010). Sequences were clustered at 95% identity and 90% coverage using CD-Hit (Fu et al., 2012). The longest sequence of each cluster was selected as a representative sequence of the cluster to create a non-redundant robust gene database. High-quality reads of the different samples were aligned to the established gene set using Bowtie2 (Langmead and Salzberg, 2012). The abundance of each gene was calculated using the method described by Song et al. (2020) and normalized by the number of mapped reads of the target gene to the gene length.

To retrieve DMSP biosynthetic and catabolic gene sequences, hidden Markov Model (HMM)-based searches for homologs in metagenome datasets were performed using HMMER tools (version3.1b2) (Eddy, 2011). Ratified DMSP biosynthetic and catabolic gene sequences were used as training sequences to create the HMM profile as described by Song et al. (2020). HMM profiles for RecA were used to estimate the proportion of bacteria in each metagenome, retrieving all hits with *E*-value ≤ 10^–50^. For DMSP biosynthetic and catabolic gene sequences, all hits with *E*-value ≤ 10^–80^ were retrieved. Approximate maximum likelihood (ML) trees for every enzyme were constructed by IQ-TREE (version 1.6.12) (Nguyen et al., 2015) under the WAG + F + G4 model with 1000 ultrafast bootstrap replicates. Putative peptide sequences that did not cluster with the clade of functionally ratified sequences were removed. To estimate the percentage of bacteria containing DMSP biosynthetic and catabolic gene sequences, the relative abundances of the curated functional enzymes were divided by the abundance of RecA retrieved in each metagenomic dataset.

## Results

### Dimethylsulfoniopropionate Content in South China Sea Sediments

As shown in Figure 1B, DMSP content in the surface sediment of SCS002–SCS011 ranged from 11.25 to 20.90 nmol g^–1^ (1 and 2 cm), and then reduced rapidly in the top 30 cm, especially between 10 and 30 cm. Our previous study (Zhang et al., 2021) revealed that oxygen was depleted rapidly in the 10-cm sediments, leading to a clear transition in microbial communities. Here, we showed that the DMSP content in sediment decreased dramatically from oxic sediments to anoxic sediments. Below 50 cm, DMSP content in the sediment remained at low levels and decreased to 0.56–2.08 nmol g^–1^ in the deepest layer of each site. Among these 10 sites, SCS005 exhibited the lowest DMSP content in the sediments, whereas SCS003, SCS011, and SCS008 showed the highest DMSP concentrations in the surface sediments compared with other sites (*p* < 0.001 in Student’s *t*-test). As shown in our previous study, SCS005 exhibited distinct TOC, C/N and δ13C profiles and different microbial community compositions, which indicated the influence of terrestrial inputs (Zhang et al., 2021). Therefore, the low DMSP content in SCS005 sediment may also be in part due to its terrestrial source, since terrestrial environments generally contain far less DMSP compared to marine sources (Yoch, 2002). In addition, sediment DMSP concentration was not correlated with the water depth. For example, although the surface sediment of SCS003 and SCS004 were obtained from similar water depth (1250 and 1260 m, respectively), the DMSP concentration in SCS003 (20.11 ± 2.62 nmol g^–1^) surface sediment was significantly higher than that of SCS004 (13.48 ± 0.81 nmol g^–1^) (*p* < 0.05 in Student’s *t*-test).

### Enrichment for Dimethylsulfoniopropionate -Producing Bacteria

**FIGURE 2:**
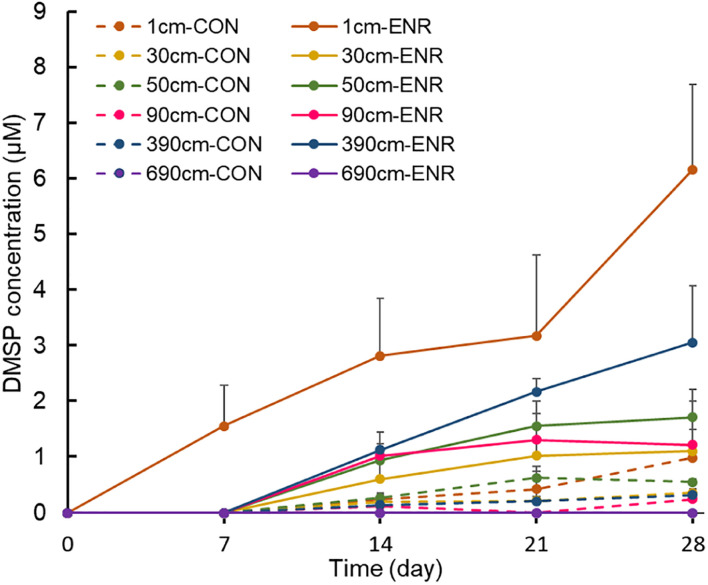
Changes of DMSP concentrations during the enrichment incubation for DMSP production. “0” means that DMSP concentration is below the detection threshold. CON, the control group in normal MBM; ENR, the DMSP-enriched groups in modified MBM.

Sediment samples from 1, 30, 50, 90, 390, and 690 cm in SCS008, which had high DMSP levels compared to the other sites, were used to enrich for and isolate DMSP-producing bacteria. The enriched cultures of surface samples showed elevated DMSP concentrations after every 7 days’ sampling event (Figure 2), and reached ∼3.5-fold higher levels (6.17 ± 1.52 μM) than those in control incubations after 28 days. In comparison, DMSP levels in the enriched cultures of subseafloor samples (30–390 cm) increased gradually only after 7 days. Surprisingly, the enriched cultures from 390-cm-deep samples showed the highest final DMSP concentrations at 28 days (3.06 ± 1.01 μM) among subseafloor samples, whereas the concentrations of enriched samples from 30, 50, and 90 cm varied between 1.11 and 1.72 μM. No DMSP was detected in the enriched cultures from the deepest layer (690 cm), even after 28 days, indicating that DMSP-producing bacteria might be too scarce to be enriched at such depth or that the incubation conditions did not favor the DMSP-producing bacteria from the deepest tested sediments.

### Quantification of Dimethylsulfoniopropionate Biosynthesis and Catabolic Genes

To study the microbial potential to synthesize DMSP within the natural sediment samples, qPCR analysis was conducted for the DMSP biosynthetic genes *dsyB* and *mmtN* (Supplementary Table 2). The abundance of *dsyB* was highest (4.95 × 10^3^ ± 1.72 × 10^3^ copies g^–1^) in the surface layer (1 cm) (Supplementary Table 2) and then reduced by ∼ an order of magnitude in deep sediments (490–790 cm). The *mmtN* gene was not detected by qPCR likely due to its low abundance in the SCS sediments. The abundance of *dsyB* was normalized by the 16S rRNA gene copies obtained in our previous study (Zhang et al., 2021), and the proportion of DMSP-producing bacteria in SCS sediment samples (containing *dsyB* only since *mmtN* cannot be detected) were predicted by qPCR. DsyB was predicted to be in 0.0007–0.0195% bacteria (Supplementary Table 2), which is far lower than previously reported predictions (0.02–3.6%) in marine sediment samples (Williams et al., 2019; Zheng et al., 2020). Note that normalization conducted in this way is not overly accurate due to the multiple copies of the 16S rRNA gene in some bacteria. Nevertheless, these results indicated that bacteria with *mmtN* and/or *dsyB* were not abundant in the SCS northern slope samples despite their DMSP stocks being significant. The reasons for this are unknown and are further investigated below through metagenomics and microbial isolation work. It is possible that the degenerate primers used here do not cover the diversity of sediment *dsyB* and/or *mmtN* genes.

To estimate bacterial DMSP catabolic activity, we performed qPCR on *dmdA* (C/2 and D/1) and *dddP*, the two most abundant DMSP catabolic genes in marine environment. These catabolic genes normally abundant in predominantly marine water samples either were undetected in all sediment depths (*dddP*) or were at very low levels that were generally (11 of 14 samples) lower than *dsyB*. The abundance of *dmdA* was highest in the top 10 cm (1.79 × 10^3^–4.27 × 10^3^ copies g^–1^) and reduced by one to two orders of magnitude below 10 cm (Supplementary Table 2). These results indicated that bacterial DMSP catabolic activity was most active in the surface sediment with high DMSP concentrations and that potentially DMSP catabolism may be less important than synthesis in these samples. It is possible that other DMSP lyase genes, not examined by qPCR here, represent the dominant catabolic genes.

**FIGURE 3:**
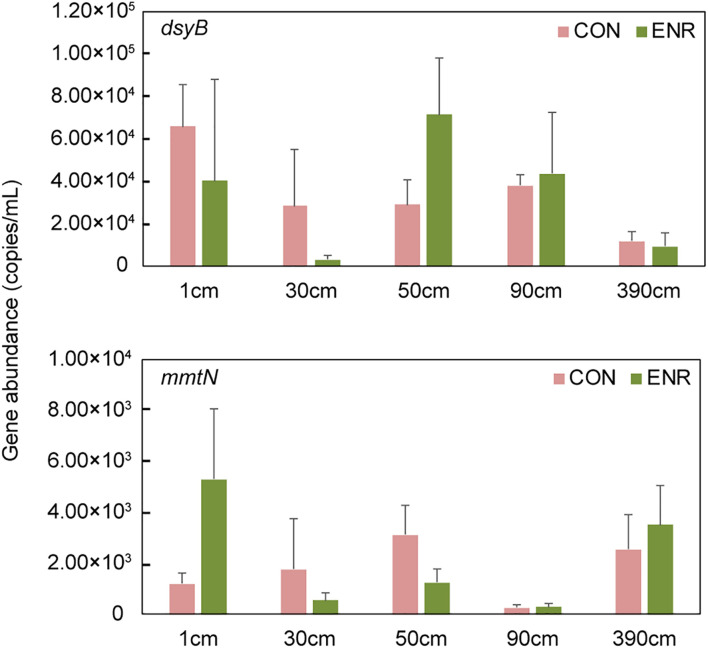
The abundance of *dsyB* and *mmtN* in the samples from enrichment incubation. CON, the control group in normal MBM; ENR, the DMSP-enriched groups in modified MBM.

Compared to the natural samples (with ∼10^2^–10^3^ copies g^–1^ *dsyB*, and *mmtN* was below detection limit), both the CON and ENR samples from the incubation experiments showed increased *dsyB* and *mmtN* abundance levels (Figure 3). However, the observed differences in the abundance of *dsyB* and *mmtN* in CON and ENR samples from all depths were not statistically significantly different (*p* > 0.05 in Student’s *t*-test), unlike DMSP levels that in most cases were far higher in the ENR samples (*p* < 0.05 in Student’s *t*-test between CON and ENR samples of 1, 30, 50, and 390 cm at 28 days) (Figure 2). Despite not being significantly more abundant, clearly higher levels of *dsyB* were detected in the ENR samples from 50 and 90 cm, and of *mmtN* in the ENR samples from the surface sediment and 390 cm, compared to CON samples (Figure 3). Besides, the abundance of *dsyB* and *mmtN* were not significantly higher in the enriched surface sediment where the DMSP concentration was the highest (*p* > 0.05 in Student’s *t*-test).

### Dimethylsulfoniopropionate-Producing Isolates in the South China Sea Sediment

**FIGURE 4:**
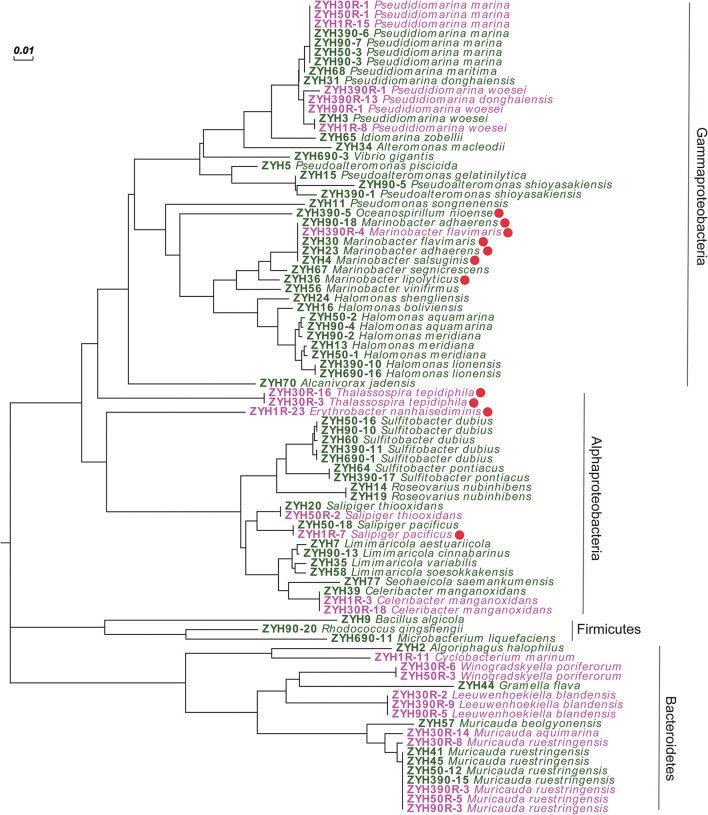
Neighbor-joining tree of 16S rRNA gene sequences of 86 representative cultivated strains. Strains isolated from the natural sediments are labeled in green, and strains isolated from the enriched sediment samples are labeled in pink. Solid red circle represents DMSP-producing isolates. Scale bar represents 0.01 substitutions per nucleotide position.

Given that our enrichment experiments highlighted the potential for novel microbial DMSP synthesis genes and/or pathways in the SCS sediment, we isolated culturable bacteria from SCS008 sediment (1, 50, 90, 390, and 690 cm), as well as ENR samples from 1, 30, 50, 90, and 390 cm, and screened them for their ability to produce DMSP. In total, 121 strains from the natural sediment (24 genera and 45 species) and 29 strains (12 genera and 15 species) from the ENR samples were isolated and identified by 16S rRNA gene sequencing (Supplementary Figure 1). Sixty and 26 representative isolates belonging to different species from the natural sediment and ENR samples, respectively, were screened for their ability to produce DMSP (Figure 4). Finally, 11 isolates belonging to *Gammaproteobacteria* and *Alphaproteobacteria* exhibited varied levels of DMSP production (Figure 4 and Supplementary Table 3), accounting for 12.8% in all representative strains. This proportion of culturable DMSP-producing isolates was lower than that previously reported in coastal sediments (25%) by Williams et al. (2019). Among these DMSP-producing isolates, only *Oceanospirillum* sp. ZYH390-5 (with *mmtN*), *Thalassospira* sp. ZYH30R-16 and ZYH30R-3 (with *mmtN*), and *Salipiger* sp. ZYH1R-7 (with *dsyB*) were shown to possess known DMSP synthesis genes by PCR with the appropriate degenerate primers.

Given *Rhodobacteraceae* bacteria are considered as important DMSP producers (Williams et al., 2019; Zhang et al., 2019), it was initially surprising that only 1 of 21 such isolates had this ability (*Salipiger* sp. ZYH1R-7). *Roseovarius* isolates of the *Rhodobacteraceae* had previously been reported to contain *dsyB* and produce DMSP (Williams et al., 2019), but isolates belonging to this genus in the current study were all incapable of DMSP biosynthesis. Furthermore, only *Salipiger* sp. ZYH1R-7 and not three other *Salipiger* isolates of the *Rhodobacteraceae* family was capable of DMSP biosynthesis. It is noteworthy that most reported DMSP-producing *Rhodobacteraceae* bacteria were isolated from seawater and, thus, this family may not be as important in these sediment environments. These data also highlight the sporadic nature of *dsyB* and *mmtN* occurrence even within genera, perhaps indicating the role of horizontal gene transfer in the prevalence of these DMSP biosynthetic genes.

Instead of *Rhodobacteraceae* bacteria, most DMSP-producing isolates were *Gammaproteobacteria* and likely contain unknown DMSP biosynthesis genes and/or pathways. Of these, *Marinobacter* strains accounted for more than half of the DMSP-producing isolates (6 out of 11), and four of them were isolated from the natural surface sediment. However, it should be noted that not all isolates in genus *Marinobacter* were able to produce DMSP under the conditions used here (e.g., strains ZYH56 and ZYH67), which is a very similar situation to the *Salipiger* and *Roseovarius* isolates described above. Gammaproteobacterial *Marinobacter* and alphaproteobacterial *Erythrobacter* strains, like those isolated here, have previously been reported as DMSP producers by Zheng et al. (2020) in work on Mariana Trench samples. These results suggested that *Marinobacter* and *Erythrobacter* bacteria might be ubiquitous DMSP producers in marine sediments and may explain why the SCS samples may be wrongly predicted to contain very low levels of DMSP-producing bacteria by qPCR work (using *dsyB* and *mmtN* primers).

### Bacterial Communities and Dimethylsulfoniopropionate Producers Revealed by 16S rRNA Gene Amplicon Sequencing

As indicated by PCoA analysis of 16S rRNA genes amplified from the SCS samples (Supplementary Figure 2), the T0 samples clustered together and are quite distinct from those generated from the incubation experiments. Furthermore, this analysis shows that the natural surface sediment community was more distinct compared with those in the subseafloor samples. Similarly, the CON and ENR samples of the surface sediment were also clearly separated from those of the subseafloor sediment where oxygen is likely limiting. Separation was also clear between the CON and ENR samples at each depth, indicating that the enrichment condition resulted in distinct microbial community composition compared to that in the CON samples.

**FIGURE 5:**
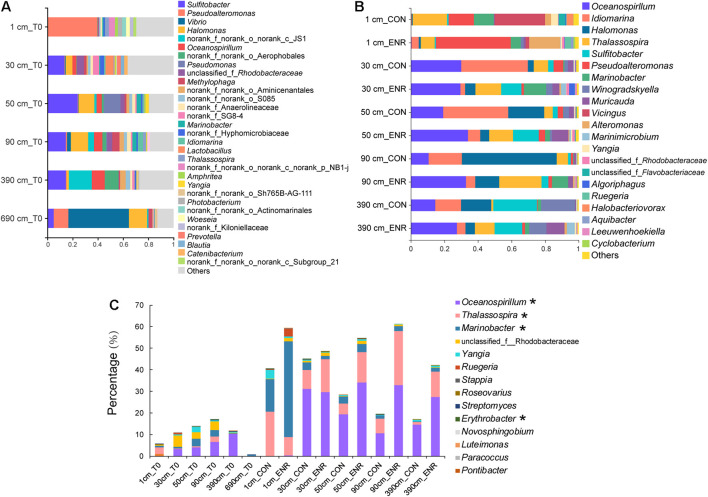
Bacterial community compositions and DMSP-producing genera in natural sediment and enriched samples revealed by 16S rRNA gene amplicon sequencing. **(A)** Bacterial communities at genera level in natural sediment samples (T0). **(B)** Bacterial communities in the control (CON) and DMSP-enriched (ENR) samples after enrichment incubation. **(C)** Known DMSP-producing genera identified from previous studies (Williams et al., 2019; Zheng et al., 2020) and this study (marked with *) in T0, CON, and ENR samples.

The natural samples from the surface sediment and the deepest layer (690 cm) contained distinct bacterial genera compared with those in samples from 30 to 390 cm, characterized by high relative abundance of *Pseudoaltermonas* and *Vibrio*, respectively (Figure 5A). The bacterial community profiles in the 30- to 390-cm sediment samples appear to be more similar, with Alphaproteobacterial *Sulfitobacter* genera dominating. Turning attention to those genera known to contain representative DMSP producers, the relative abundance of these bacteria was higher in the 30-, 50-, 90-, and 390-cm samples than those from surface sediment but was by far the lowest in the 690-cm samples (Figure 5C). This contradicts the qPCR absolute abundance data that showed *dsyB* to be more abundant in the surface sediment (Supplementary Table 2). *Oceanospirillum*, *Thalassospira*, *Marinobacter*, and *Rhodobacteraceae* species tended to be the dominant predicted DMSP-producing groups in the SCS subseafloor (Figure 5C). Few predicted DMSP-producing taxa were found at 690 cm, and this might explain the failure of enrichment experiment for DMSP producers at this depth (Figure 5C). We found no plastid sequences in the surface sediment and only few sequences belonging to *Bacillariophyta*, Chlorophyta, and *Streptophyta* in deep sediments (Supplementary Table 4), accounting for 0–0.06% of the community sequences. The proportion of eukaryotic plastid was far lower than that has been reported in the Stiffkey saltmarsh sediments (9%) (Williams et al., 2019). Considering the extremely low abundance of eukaryotic algae and that most were likely dead in such dark deep-sea sediment, we propose that these eukaryotic DMSP producers were not likely important DMSP producers in these sediments.

**FIGURE 6:**
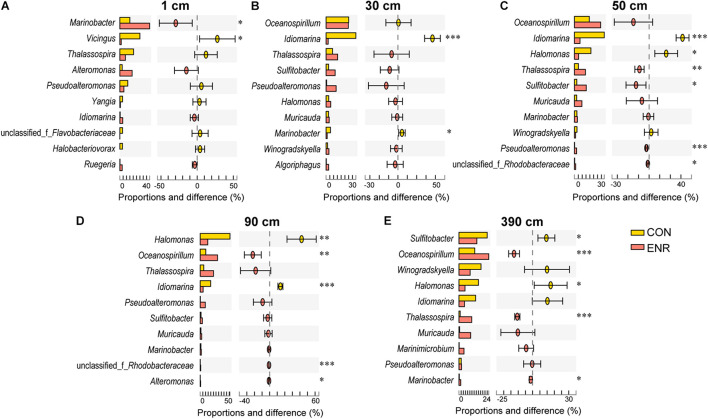
Genus-level differences between the control and DMSP-enriched samples. The proportion represents the relative abundance of each genera revealed by 16S rRNA gene amplicon sequencing. CON, the control group in normal MBM; ENR, the DMSP-enriched groups in modified MBM. *p < 0.01 in Student’s *t*-test; **p < 0.001 in Student *t*-test; ***p < 0.0001 in Student’s *t*-test.

In the incubation experiments designed to enrich for DMSP production, we observed that the enrichment conditions of high salt, low nitrogen conditions and the supplementation of L-Met, led to clear changes in bacterial communities (Figure 5B). This is consistent with those genera more abundant in the ENR samples potentially being DMSP producers. Furthermore, distinct bacterial groups were enriched from the surface and subseafloor sediment experiments. The ENR samples from all depths consistently showed higher proportions of predicted DMSP-producing genera increased by 6.6–42.2% compared with the corresponding CON samples (Figure 5C). *Marinobacter* (a genus without known DMSP synthesis genes) was the most significantly enriched genera in the ENR samples of the surface sediment (Figure 6A). Conversely, *Thalassospira* that can contain MmtN (Williams et al., 2019) was most enriched in the ENR samples from 30, 50, 90, and 390 cm, and *Oceanospirillum* was enriched in those of 50, 90, and 390 cm (Figures 6B–D). Several *Rhodobacteraceae* species were also enriched in the ENR samples from 50 and 90 cm (Figures 6C,D). In contrast, *Idiomarina*, a genus not known to contain DMSP-producing representatives yet, was more abundant in the CON groups of the subseafloor samples. The genus *Halomonas* was more abundant in the CON samples of 50, 90, and 390 cm than in the ENR samples.

### Dimethylsulfoniopropionate Biosynthesis Gene Revealed by Metagenomic Analysis

**FIGURE 7:**
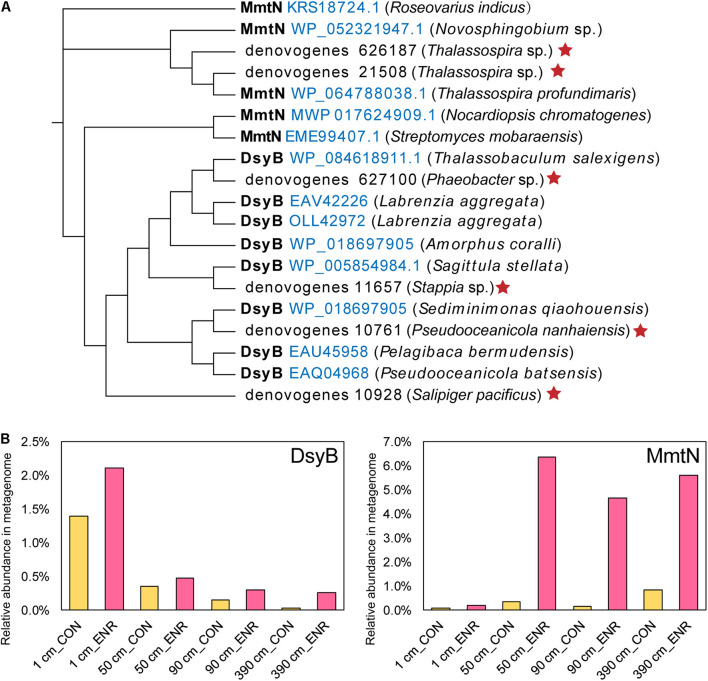
DMSP synthesis proteins predicted from metagenomic analysis of enriched cultures. **(A)** Maximum-likelihood phylogenetic tree of DsyB and MmtN sequences from metagenomes (indicated by red stars) and functionally ratified proteins (with the Accession Number in blue). **(B)** The relative abundance of DMSP biosynthesis protein sequences in metagenomes. CON, the control group in normal MBM; ENR, the DMSP-enriched groups in modified MBM.

To further identify known DMSP biosynthesis and catabolic gene sequences and their abundance in the incubation experiments, we performed metagenomic sequencing on the CON and ENR samples of 1, 50, 90, and 390 cm. Two MmtN sequences and four DsyB sequences were retrieved from the metagenomic data, clustering with ratified functional MmtN or DsyB (Figure 7A). The MmtN sequences belonged to *Thalassospira* species, and DsyB sequences were closely resembled sequences from *Rhodobacteraceae* including *Phaeobacter*, *Stappia*, *Pseudooceanicola*, and *Salipiger*. The relative abundance of MmtN and DsyB increased in all ENR samples (Figure 7B) compared with the CON samples, confirming the effectiveness of the enrichment conditions. The highest relative abundance of *dsyB* was found in the ENR samples of the surface sediments (2.11%), and the abundance of dsyB in both CON and ENR samples was reduced with increased sediment depth. In contrast, *mmtN* was more highly enriched in the ENR samples of the subseafloor sediments (50, 90, and 390 cm), reaching high proportions of 4.65–6.36%, than in the surface samples. These results indicated that the dominant known DMSP biosynthesis gene in the ENR samples of surface sediment was *dsyB*, but the unknown DMSP biosynthesis genes from *Marinobacter* may eclipse this when and if it is identified. In contrast, the metagenomics data suggest *mmtN* as the dominant DMSP synthesis gene in the ENR subseafloor samples. We also investigated the changes in relative abundance of DMSP catabolic genes during the incubation experiments (Supplementary Table 5). In general, possibly due to the increased DMSP concentrations, the relative abundance of the major DMSP catabolic gene *dmdA* was considerably higher in the ENR samples. In most cases, DMSP lyase genes were not more abundant in the ENR samples, the exceptions being *dddD* and *dddQ* in the surface sediment (Supplementary Table 5). This resulted in a higher overall percentage of total bacterial DMSP catabolic than synthesis genes in most ENR samples, except for that at 390 cm, where the abundance of *dddL* was extremely higher in the CON samples.

## Discussion

As an important compound involved in global sulfur cycling, the production of DMSP has been widely studied in the euphotic layers of marine systems, with varied concentrations ranging from nanomolar to micromolar (Zhang et al., 2019). It is estimated that about 8 billion tons of DMSP is produced by phytoplankton annually in the Earth’s surface oceans (Galí et al., 2015), and DMSP concentration generally decreases with water depth, likely due to the reduced abundance of phytoplankton (Zheng et al., 2020). However, multiple studies revealed that DMSP and DMS concentrations in marine sediment reach ∼1,000-fold higher levels than those in the overlying water column (Nedwell et al., 1994; Wilkening et al., 2019; Williams et al., 2019), suggesting that marine surface sediments are hot spots for DMSP production and catabolism. In these listed studies, surface sediment DMSP concentrations are very high, e.g., ∼100 nmol g^–1^ sediment in the coastal sediments (Williams et al., 2019) and ∼600 nmol g^–1^ in a sulfidic pond of Warham marsh (Wilkening et al., 2019). A portion of this DMSP in the photic surface sediments may originate from algae, e.g., in a diatom-dominated intertidal sediment with 100–150 nmol g^–1^ (Van Bergeijk et al., 2002). In a study of deep sea sediment, DMSP concentration tended to be lower, ranging 3.15–6.14 nmol g^–1^ (Zheng et al., 2020) and 0.1–6 nmol g^–1^ in the North Sea (Nedwell et al., 1994), but the contribution of phytoplankton here is likely less significant. The DMSP concentration range in the SCS sediment (11.25–20.90 nmol g^–1^) may represent typical DMSP content in continent slope environments, falling in between those of the deep-sea sediments and coastal sediments. Our results indicated that the DMSP concentration in surface sediment was not directly correlated with the water depth; other issues, such as terrestrial inputs, may exert more influence on DMSP concentrations in marine sediments.

In the current study, we delineated a detailed depth profile of DMSP concentration in sediment down to 790 cm. Consistent with previous studies on salt marsh sediment (Wilkening et al., 2019; Williams et al., 2019), the DMSP concentration reduced dramatically between 10 and 30 cm, but DMSP levels remained at a detectable level even at 790 cm, indicating that there might be DMSP-producing and -consuming bacteria buried deep in subseafloor sediments. A significant limitation of this study is that we were unable to carry out experiments to study DMSP synthesis and catabolic rates. Therefore, it should be noted that the DMSP stocks reported here in the SCS sediment do not necessarily reflect synthesis activity, if in the case that DMSP turnover is insignificant.

The abundance of eukaryotic algae in the SCS sediments was extremely low as indicated by the plastid sequences in 16S rRNA gene amplicon sequencing data, and the DMSP biosynthesis activity of these eukaryotic algae are yet to be proved, especially in the subseafloor, suggesting that bacteria might be the major DMSP producers in sediments. The *dsyB* and *mmtN* are considered as robust reporters for bacterial DMSP biosynthesis in diverse marine environment (Curson et al., 2017; Williams et al., 2019). The abundance of *dsyB* can reach as high as 10^7^ copies g^–1^ in coastal sediments (Williams et al., 2019; Liu et al., 2021), and is generally always more abundant than *mmtN*, e.g., *mmtN* is ∼four orders of magnitude less abundant than *dsyB* in different sediment samples (Williams et al., 2019; Zheng et al., 2020; Liu et al., 2021). However, the highest abundance of *dsyB* detected in the SCS sediment was only 10^3^ copies g^–1^, and *mmtN* were below the detection limit by qPCR. These values were even lower than that in the deep-sea sediments of the Mariana Trench (10^5^ copies g^–1^ of *dsyB* and 10^2^ copies g^–1^ of *mmtN*) (Zheng et al., 2020), although the DMSP concentrations in the SCS sediment were higher than that in the Mariana Trench sediment. These results suggested that there could be novel DMSP-producing bacteria in SCS sediment with unidentified DMSP biosynthesis gene that contributed to the DMSP content. Indeed, only 4 out of 11 DMSP-producing bacterial isolates obtained from the SCS sediment possessed *dsyB* or *mmtN* (Supplementary Table 3), and the genes involved in DMSP biosynthesis in, e.g., *Marinobacter* and *Erythrobacter* are yet to be determined. We hypothesize that these unknown genes encode major DMSP synthesis systems in the SCS samples examined here. Despite the low abundance of known DMSP biosynthesis genes in the SCS sediment, the 16S rRNA gene amplicon sequencing revealed distinct profiles of known DMSP-producing genera at different depths (Figure 5C). At 690 cm, few known DMSP-producing taxa were identified, and this may indicate that this depth may represent a point below which microbial DMSP synthesis is not favorable; thus, only very few DMSP-producing bacteria can persist at such depths.

We also quantified the abundance of bacterial DMSP catabolism genes (mainly *dddP* and *dmdA* by qPCR) in the SCS sediments. Bacteria with the ability to catabolize DMSP *via dmdA* and *ddd* genes are generally extremely high in seawater samples (Moran and Durham, 2019; Zhang et al., 2019), much higher than those potentially synthesizing DMSP *via dsyB* and *mmtN* (Curson et al., 2018; Williams et al., 2019). Interestingly, bacterial DMSP catabolism genes were detected at much lower than typical surface seawater levels that were very similar to those of the DMSP biosynthesis genes, despite DMSP sediment stocks being far higher than that in seawater. The low predicted abundance of DMSP catabolic bacteria in SCS sediment may be directly responsible for accumulation and thus the high DMSP stocks observed in these sediments. It is also possible that the qPCR primers used are not detecting deep sediment variants of DMSP catabolic genes, or that other DMSP catabolic enzymes (not *dmdA* or *dddP*) and/or isoform enzymes exist in sediments. Further work focused on bacterial DMSP catabolism genes; their expression and process rates are required to better establish the importance of bacteria in DMSP cycling throughout the sediment depth profile, and to test the hypotheses raised here.

As in other previous studies (Williams et al., 2019; Liu et al., 2021), the incubation experiments conducted here have been proved as an effective method to enrich for DMSP-producing bacteria, and increased DMSP concentrations were detected in the ENR samples. As indicated by the 16S rRNA gene amplicon sequencing results, several DMSP-producing genera were significantly enriched in the ENR samples compared with the CON samples (Figures 5B,C). In addition, differences existed between the enriched bacterial groups from the surface and subseafloor sediments (Figures 5, 6); the significantly enriched DMSP-producing group was most *Marinobacter* in the surface sediment, whereas the enriched subseafloor samples were typified by increased abundance of *Oceanospirillum* and *Thalassospira*. Although no significant differences of *dsyB* and *mmtN* abundance between CON and ENR samples were detected by qPCR, the metagenomic data suggested that the percentage of *dsyB*- and *mmtN*-containing bacteria increased in all the enriched samples. Interestingly, *dsyB* accounted for higher relative abundance in the enriched surface sediment, while the abundance of *mmtN* were much higher in the enriched subseafloor sediment samples. These results also suggested that distinct DMSP-producing bacteria existed in surface and subseafloor sediments.

Dominant DMSP-producing bacteria in the SCS sediment were from *Thalassospira*, *Oceanospirillum*, and *Rhodobacteraceae*, as well as several genera with unknown DMSP biosynthesis genes/pathways, such as *Marinobacter* (Figure 5C). *Marinobacter* species accounted for a considerable proportion in the enriched surface sample, as well in the DMSP-producing isolates we obtained. Our results emphasized the prevalence of this novel DMSP-producing group, especially in the marine sediment. Another important DMSP-producing isolate we obtained was *Erythrobacter* sp. ZYH1R-23. Although *Erythrobacter* was not a dominant DMSP-producing genus in either natural and enriched samples, ZYH1R-23 showed high DMSP-producing capacity compared to most other DMSP-producing isolates (Supplementary Table 3). We also noticed that within the same genus, some isolates were capable of DMSP production while others were not, as for *Marinobacter*, *Roseovarius*, and *Salipiger*. Therefore, although predicting DMSP-producing bacteria at the genus level is the most direct way to go about variations in DMSP-producing groups, it may exaggerate the proportions of DMSP biosynthesis bacteria when estimated at the genus level, and these results needed to be treated with caution. Additionally, it is interesting to further investigate the heterogeneity of DMSP production within the same genus considering the source and genetics of different strains, and to identify the novel DMSP synthesis genes and/or pathways in DMSP-producing bacteria like *Marinobacter* and *Erythrobacter* that lack *dsyB* and *mmtN*. Finally, it is important to note that the bacterial isolation work and incubation experiments conducted here were done under aerobic conditions which will select only for facultative anaerobes in the subseafloor anerobic sediment samples. Thus, further work is required to establish if any obligate anaerobes synthesize DMSP and how they may do so. This is potentially an important topic for future research.

## Conclusion

The observed DMSP content in SCS sediment may represent typical levels for continent slope environments. These DMSP levels clearly decreased from the surface to deep subseafloor sediments. Distinct DMSP biosynthesis bacteria existed in the surface and subseafloor sediments. The abundance of bacterial DMSP biosynthesis and catabolism genes decreased from the oxic sediment to anoxic sediment. In addition to *dsyB*- and *mmtN*-containing bacteria, novel DMSP-producing bacteria with unknown DMSP biosynthesis gene were identified in the SCS sediments, such as *Marinobacter* and *Erythrobacter*. The abundance of these DMSP-producing groups increased during the enrichment incubation experiments. Further work is needed to identify the DMSP biosynthesis pathway and key genes in these novel DMSP-producing bacteria.

## Data Availability Statement

The datasets presented in this study can be found in online repositories. The names of the repository/repositories and accession number(s) can be found in the article/Supplementary Material.

## Author Contributions

X-HZ, JT, and YZ conceived the study. YZ collected samples, performed experiments, analyzed data, and wrote the manuscript. KS and CS performed isolation and identification of DMSP-producing strains. XS performed sampling. All authors edited and approved the manuscript.

## Conflict of Interest

The authors declare that the research was conducted in the absence of any commercial or financial relationships that could be construed as a potential conflict of interest.

## Publisher’s Note

All claims expressed in this article are solely those of the authors and do not necessarily represent those of their affiliated organizations, or those of the publisher, the editors and the reviewers. Any product that may be evaluated in this article, or claim that may be made by its manufacturer, is not guaranteed or endorsed by the publisher.

## Footnotes

1
http://www.ezbiocloud.net/identify

2
http://drive5.com/uparse/

3
http://rdp.cme.msu.edu/

4
http://www.arb-silva.de
