# Supplementary material for: Dimethylsulfoniopropionate Biosynthetic Bacteria in the Subseafloor Sediments of the South China Sea
Source: Front Microbiol. 2021 Oct 11;12:731524. doi: 10.3389/fmicb.2021.731524 (PMC8543147; doi:10.3389/fmicb.2021.731524)
Supplement: Supplementary file 1 [file Table_1.DOCX]

Supplementary Material

*Supplementary Tables*

Supplementary Table 1. Primers and amplification conditions for qPCR.

| Target gene | Primer Sequence (5’-3’) | Amplicon length (bp) | Annealing temperature (°C) |
| --- | --- | --- | --- |
| *dsyB* | dsyBF: CATGGGSTCSAAGGCSCTKTT  dsyBR: GCAGRTARTCGCCGAAATCGTA | 246 | 61 |
| *mmtN* | mmtNF: CCGAGGTGGTCATGAAYTTYGG  mmtNR: GGATCACGCACACYTCRTGRTA | 301 | 54 |
| *dddP* | 874F: AAYGAAATWGTTGCCTTTGA  971R: GCATDGCRTAAATCATATC | 97 | 41 |
| *dmdA* (C/2) | 291F: AGATGAAAATGCTGGAATGATAAATG  482R: AAATCTTCAGACTTTGGACCTTG | 191 | 50 |
| *dmdA* (D/1) | 268F: AGATGTTATTATTGTCCAATAATTGATG  356R: ATCCACCATCTATCTTCAGCTA | 89 | 49 |

Supplementary Table 2. The abundance of *dsyB* and *mmtN* in the SCS sediment quantified by qPCR.

| Depth (cm) | Gene abundance (copies/g) | | | | | | | Percentage (%) | |
| --- | --- | --- | --- | --- | --- | --- | --- | --- | --- |
|  | *dsyB* | *mmtN* | *dmdA*-D/1 | *dmdA*-C/2 | *dddP* | 16S rRNA gene* | Biosynthetic gene | | Catabolic gene |
| 1 | 4.95×10^3^ ± 1.72×10^3^ | ND | 4.78×10^1^±9.60×10^0^ | 1.74×10^3^±3.09×10^2^ | ND | 1.43×10^8^ | 0.0035% | | 0.0012% |
| 2 | 1.14×10^3^ ± 4.56×10^2^ | ND | 2.06×10^2^±3.37×10^1^ | 4.25×10^3^±4.90×10^2^ | ND | 1.68×10^8^ | 0.0007% | | 0.0026% |
| 6 | 2.57×10^3^ ± 2.17×10^3^ | ND | 4.11×10^3^±2.13×10^2^ | 2.75×10^2^±3.03×10^1^ | ND | 5.22×10^7^ | 0.0049% | | 0.0084% |
| 10 | 1.03×10^3^ ± 4.70×10^2^ | ND | 2.69×10^3^±1.08×10^3^ | 3.88×10^2^±7.97×10^1^ | ND | 3.56×10^7^ | 0.0029% | | 0.0086% |
| 30 | 1.01×10^3^ ± 7.17×10^2^ | ND | 1.57×10^2^±8.08×10^1^ | ND | ND | 6.94×10^6^ | 0.0146% | | 0.0023% |
| 50 | 2.02×10^3^ ± 1.24×10^3^ | ND | 1.35×10^2^±6.00×10^1^ | 7.67×10^1^±2.46×10^1^ | ND | 3.81×10^7^ | 0.0053% | | 0.0006% |
| 90 | 2.01×10^3^ ± 5.59×10^2^ | ND | 2.49×10^1^±1.23×10^1^ | ND | ND | 2.43×10^7^ | 0.0083% | | 0.0001% |
| 190 | 1.25×10^3^ ± 4.14×10^2^ | ND | 1.17×10^2^±3.59×10^1^ | 1.37×10^1^±4.97×10^0^ | ND | 1.04×10^7^ | 0.0120% | | 0.0013% |
| 290 | 1.44×10^3^ ± 1.70×10^2^ | ND | 1.90×10^2^±1.35×10^2^ | 9.32×10^1^±3.92×10^1^ | ND | 5.18×10^7^ | 0.0028% | | 0.0005% |
| 390 | 1.58×10^3^ ± 5.48×10^2^ | ND | 3.07×10^1^±2.08×10^1^ | ND | ND | 1.06×10^7^ | 0.0149% | | 0.0003% |
| 490 | 3.12×10^2^ ± 9.27×10^1^ | ND | 2.96×10^1^±5.22×10^0^ | 9.57×10^1^±2.09×10^0^ | ND | 8.57×10^6^ | 0.0036% | | 0.0015% |
| 590 | 7.79×10^2^ ± 3.38×10^2^ | ND | 1.35×10^2^±7.14×10^0^ | ND | ND | 5.61×10^6^ | 0.0139% | | 0.0024% |
| 690 | 9.10×10^2^ ± 1.60×10^2^ | ND | 1.00×10^2^±3.90×10^1^ | 7.93×10^1^±6.45×10^1^ | ND | 4.66×10^6^ | 0.0195% | | 0.0038% |
| 790 | 1.42×10^2^ ± 7.96×10^2^ | ND | 1.26×10^2^±3.29×10^0^ | 1.72×10^1^±1.61×10^0^ | ND | 3.85×10^6^ | 0.0037% | | 0.0037% |

ND, could not be determined due to the low abundance.

*, data from previous study by Zhang et al. (2021).

Supplementary Table 3. DMSP biosynthesis isolates in SCS sediments.

| Isolates | Taxonomy | Source | DMSP production in normal MBM (μM) | DMSP biosynthesis gene |
| --- | --- | --- | --- | --- |
| ZYH4 | *Marinobacter salsuginis* | T0 | 1.15 ± 0.05 | ND |
| ZYH23 | *Marinobacter adhaerens* | T0 | 0.10 ± 0.05 | ND |
| ZYH30 | *Marinobacter flavimaris* | T0 | 0.90 ± 0.03 | ND |
| ZYH36 | *Marinobacter lipolyticus* | T0 | 0.31 ± 0.06 | ND |
| ZYH90-18 | *Marinobacter adhaerens* | T0 | 0.39 ± 0.04 | ND |
| ZYH390-5 | *Oceanospirillum nioense* | T0 | 0.10 ± 0.03 | *mmtN* |
| ZYH1R-23 | *Erythrobacter nanhaisediminis* | ENR | 4.02 ± 0.16 | ND |
| ZYH1R-7 | *Salipiger pacificus* | ENR | 8.74 ± 0.18 | *dsyB* |
| ZYH30R-16 | *Thalassospira tepidiphila* | ENR | 1.72 ± 0.10 | *mmtN* |
| ZYH30R-3 | *Thalassospira tepidiphila* | ENR | 0.44 ± 0.02 | *mmtN* |
| ZYH390R-4 | *Marinobacter flavimaris* | ENR | 1.04 ± 0.04 | ND |

ND, not detected by PCR with degenerate primer of *dsyB* and *mmtN*.

Supplementary Table 4. Plastid sequences in 16S rRNA gene sequencing data.

| Depth (cm) | Number of sequences | | |
| --- | --- | --- | --- |
|  | Bacillariophyta | Chlorophyta | Streptophyta |
| 1 | 0 | 0 | 0 |
| 2 | 1 | 0 | 0 |
| 6 | 1 | 0 | 0 |
| 10 | 0 | 0 | 0 |
| 30 | 3 | 0 | 1 |
| 50 | 0 | 2 | 3 |
| 90 | 0 | 0 | 0 |
| 190 | 0 | 0 | 0 |
| 290 | 1 | 0 | 0 |
| 390 | 6 | 0 | 0 |
| 490 | 2 | 0 | 1 |
| 590 | 3 | 0 | 17 |
| 690 | 0 | 0 | 0 |
| 790 | 3 | 0 | 21 |

Supplementary Table 5. The relative abundance of DMSP catabolism protein sequences in metagenomes.

|  | 1cm_CON | 1cm_ENR | 50cm_CON | 50cm_ENR | 90cm_CON | 90cm_ENR | 390cm_CON | 390cm_ENR |
| --- | --- | --- | --- | --- | --- | --- | --- | --- |
| DmdA | 0.8235% | 1.9206% | 5.9754% | 11.5986% | 2.5741% | 5.5800% | 1.5841% | 4.5357% |
| DddP | 0.9015% | 0.1562% | 0.1487% | 0.0000% | 0.0466% | 0.0102% | 0.0632% | 0.0105% |
| DddD | 0.2643% | 4.6126% | 0.5906% | 0.4085% | 0.0727% | 0.0748% | 0.4642% | 0.3502% |
| DddL | 0.9068% | 1.4030% | 0.6180% | 0.4734% | 1.1798% | 0.1630% | 21.9715% | 3.9356% |
| DddQ | 0.0273% | 0.1082% | 0.0000% | 0.0000% | 0.0000% | 0.0000% | 0.0000% | 0.0000% |
| DddW | NF | NF | NF | NF | NF | NF | NF | NF |
| DddY | NF | NF | NF | NF | NF | NF | NF | NF |
| DddK | NF | NF | NF | NF | NF | NF | NF | NF |
| Total | 2.9234% | 8.2005% | 7.3328% | 12.4805% | 3.8732% | 5.8279% | 24.0831% | 8.8319% |

NF, not found in the metagenomes

*Supplementary Figures*


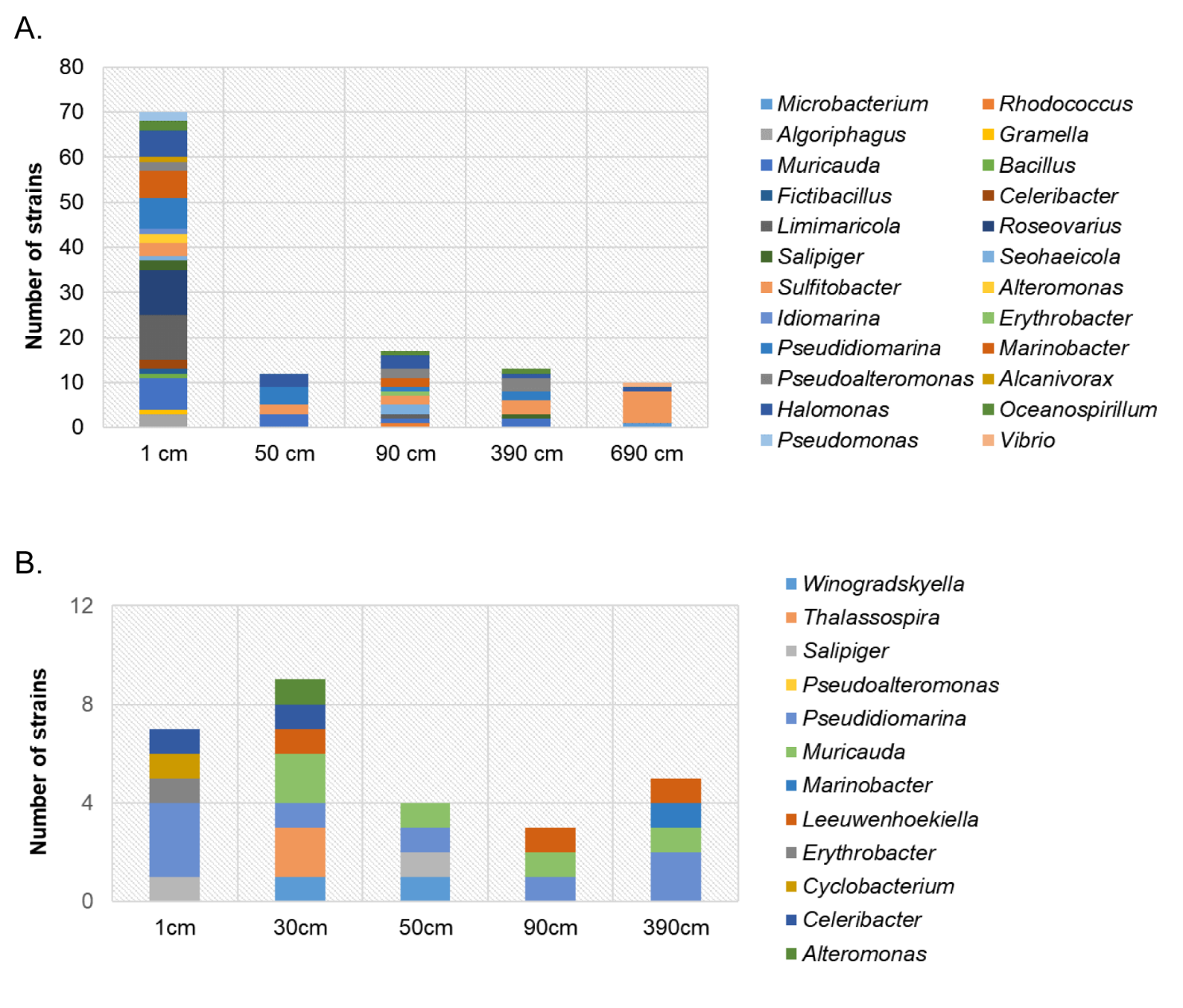


Supplementary Figure 1. Culturable strains isolated from the SCS natural sediments and the enriched samples.


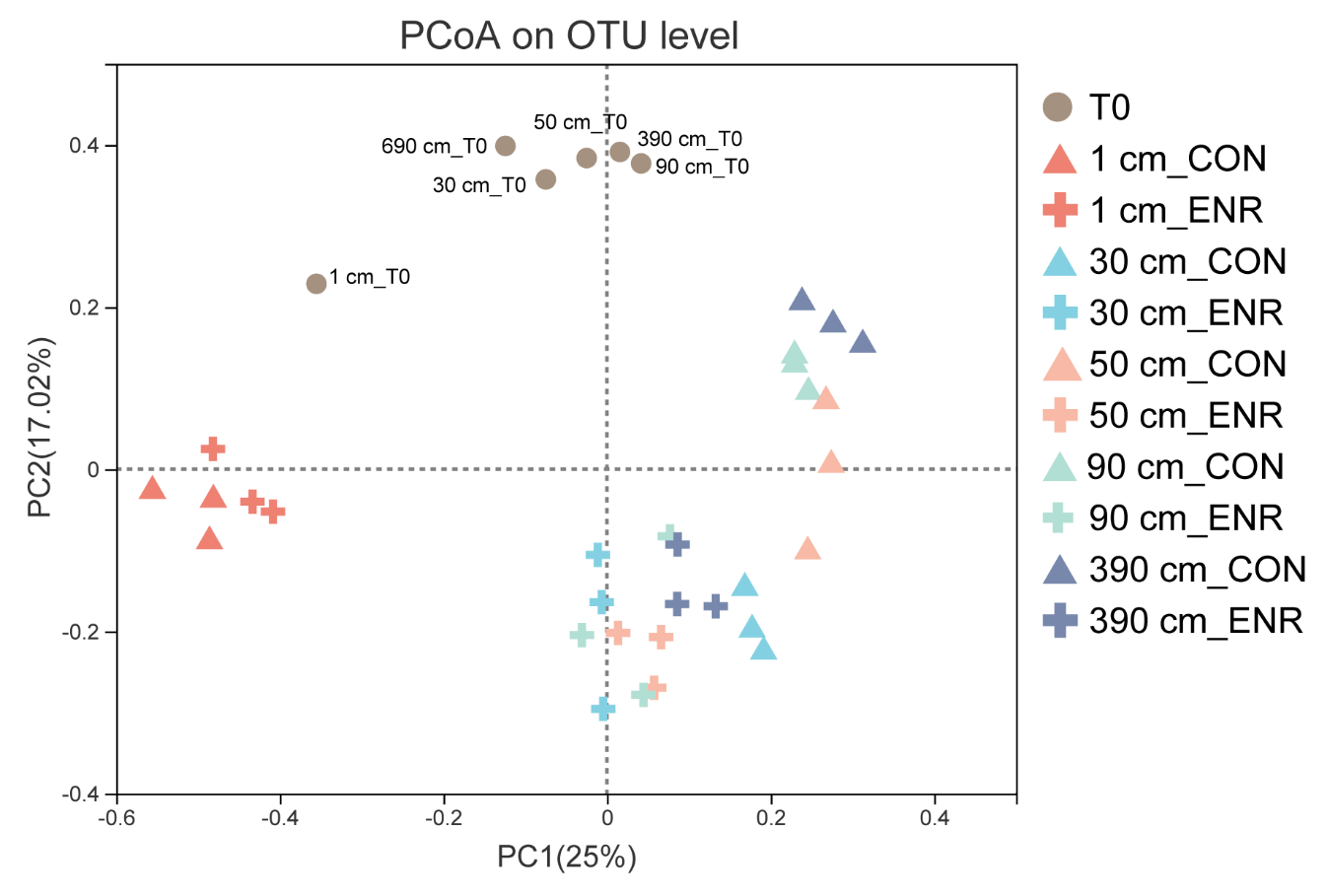


Supplementary Figure 2. PCoA analysis on natural sediment and samples from enrichment incubation experiments. T0, natural sediment samples; CON, the control group in normal MBM; ENR, the DMSP-enriched groups in modified MBM.
